# Editorial: Epilepsy research in the developing countries: the neuro-ethical issues, cross-cultural differences, and relevance to comprehensive management

**DOI:** 10.3389/fneur.2024.1429745

**Published:** 2024-05-23

**Authors:** Mark Kaddu Mukasa, Albert Akpalu, Adetoun Ejilemele, Olubunmi Akindele Ogunrin

**Affiliations:** ^1^Neurology Unit, Department of Medicine, Makerere University, Kampala, Uganda; ^2^Neurology Unit, Korle Bu Teaching Hospital, Accra, Ghana; ^3^Department of Pathology and Immunology, Texas Children's Hospital/Baylor College of Medicine, Houston, TX, United States; ^4^Neurology Department, Royal Stoke University Hospital, Stoke-on-Trent, United Kingdom

**Keywords:** epilepsy, neuroethics, cross-cultural differences, developing countries, management, epilepsy surgery

Epilepsy, defined as the sustained predisposition to recurrent seizures with neurobiological, cognitive, social and psychological sequelae, remains the most common disorder encountered in neurological practice (1–3). The often-quoted prevalence of 50 million worldwide is likely inaccurate considering the lack of complete data on epilepsy from developing countries. However, in the past decade and a half, the extant literature that originated from these countries, provided information on the current trends in epilepsy management and scope of epilepsy research (4, 5). An updated and balanced understanding of the impact of epilepsy research on holistic health of affected individuals, including cognitive sequelae, cross-cultural differences in the management, social support, and the ethical, legal, and social issues (ELSI) encountered in epilepsy research are topical and demand our attention but are rarely discussed. Therefore, this volume aims to provide information to bridge these knowledge gaps as they relate to the low- and medium-income countries (LMICs) and stimulate valid and ethically acceptable epilepsy research across cultures and disciplines.

Eight articles from diverse cultures and settings addresses this Research Topic in this volume of *Frontier in Neurology*. One of the articles is an interesting qualitative study that focused on perceived etiology and management of epilepsy among a rural West African community in Ghana (Gyaase et al.). Using an in-depth semi-structured interview, data obtained from 15 participants revealed two causative categories for epilepsy—socio-cultural (mostly related to superstitious beliefs), and biomedical. Authors recommend comprehensive health education to promote knowledge on causative factors and treatment of epilepsy. Several studies have reported relationship between superstitious beliefs, causation, and management of epilepsy (6–10). Absence of knowledge on causative factors of epilepsy leads to delay in treatment and worsens outcomes in LMIC's.

Li et al. describe a nested case-control study exploring the effect of epilepsy education shared during the epilepsy knowledge popularization campaign in China (EKPCIC) on pregnancy outcomes. Their study showed that there was an increase in the use of anti-seizure medicine (ASM), folic acid, breastfeeding rate as well as fewer pregnancy complications. Adverse fetal outcomes were however not affected.

Genetic causes are often not considered in LMIC's but etiology of epilepsy is multifactorial often with an interplay between genetics and environment. Xu et al., investigated the role of ferroptosis-related genes in the pathogenesis of epilepsy using the CIBERSORT algorithm analysis and screening gene expression data from the Gene Expression Omnibus (GEO) database. Three ferroptosis related differentially expressed genes (FDEGs)—HIF1A, TLR4, and CASP8 were identified and authors recommend mechanistic studies be performed to investigate the pathways modulated by these genes and their role in epilepsy.

The social impact of epilepsy is not often discussed in LMICs (11, 12) presumably due to patriarchy and gender inequality. Two studies investigated the social and psychological sequelae of epilepsy. One article evaluated marriage and child bearing among PWEs in a hospital population in Turkey (Tokuç et al.). The authors showed that a third of the PWEs experienced stigmatization and that epilepsy diagnosis impacted sexual function adversely. Furthermore, epilepsy diagnosis led to a lack of desire to have children and the ability to care for the children was adversely affected by the use of multiple ASM and female gender. The findings of this study illustrate the scope of impact of epilepsy on the overall quality of life of affected persons, as previously revealed in a few studies (13–15). A separate article studied stress-related symptoms among PWEs in Ethiopia and showed that nearly one out of 4 PWEs has stress symptoms, and that perceived stigma and belief that epilepsy was untreatable were associated factors (Seid et al.).

Pharmacotherapy is the mainstay of epilepsy treatment, and ~60–70% PWEs respond to ASM with 50–60% responding to monotherapy within the first 6 months (16–18). Refractory epilepsy requiring other therapies to improve or achieve seizure control is present in 20–30% (19). Two articles discussed epilepsy management with one focused on drug treatment with Perampanel among Chinese PWEs (Zeng and Wu). Perampanel was more effective in treating focal seizures and was well-tolerated when used as first option in individuals who had failed the first ASM. The most common adverse event leading to discontinuation of medication was aggression and irritability.

Epilepsy surgery is gradually evolving in Africa, with majority of centers performing lesional surgeries. The Armenian article (Sukhudyan et al.) recorded significant seizure control in ninety percent of patients proving that the model in a middle income country can motivate other LMICs to develop epilepsy surgery.

The final article by Samia et al. addressed the ethical and validity conundrum in epilepsy research in LMICs, a core aspect of this Research Topic. They highlighted the heterogeneous settings of LMICs as relates to endemic infections and metabolic diseases and its impact on epilepsy research. They advocated for support for researchers and other stakeholders involved with epilepsy research, not just through provision of funds but more importantly by ensuring a foundation for ethical template of equality, capacity building and intellectual property protection. They suggested provision of culturally relevant and responsive ethical framework to promote valid research in the LMICs.

## Author contributions

OO: Conceptualization, Project administration, Supervision, Validation, Writing – original draft, Writing – review & editing. MK: Writing – review & editing, Validation. AA: Writing – review & editing, Validation. AE: Writing – review & editing, Validation.

## References

[1] Ba-Diop A Marin B Druet-Cabanac M Ngoungou EB Newton CR Preux PM. Epidemiology, causes, and treatment of epilepsy in sub-Saharan Africa. Lancet Neurol. (2014) 13:1029–44. 10.1016/S1474-4422(14)70114-0 25231525 PMC5497080

[2] Prevett M. Epilepsy in sub-Saharan Africa. Pract Neurol. (2013) 13:14–20. 10.1136/practneurol-2012-000388 23315455

[3] Stafstrom CE Carmant L. Seizures and epilepsy: an overview for neuroscientists. Cold Spring Harb Perspect Med. (2015) 5:a022426. 10.1101/cshperspect.a022426 26033084 PMC4448698

[4] Preux P-M Druet-Cabanac M. Epidemiology and aetiology of epilepsy in sub-Saharan Africa. Lancet Neurol. (2005) 4:21–31. 10.1016/S1474-4422(04)00963-9 15620854

[5] Olubunmi A Ogunrin OA. Epilepsy in Nigeria—a review of etiology, epidemiology and management. Benin J Postgrad Med. (2006) 8:27–51. 10.4314/bjpm.v8i1.47362

[6] Mameniškiene R Kizlaitiene R Kaladyte Lokominiene R Puteikis K. Belief in omens and superstitions among patients with chronic neurological disorders. Front Publ Health. (2024) 12:1331254. 10.3389/fpubh.2024.1331254 38525335 PMC10958788

[7] Ogunrin OA Obiabo OY Obehigie E. Risk factors for epilepsy in Nigerians-a cross-sectional case-control study. Acta Neurol Scand. (2014) 129:12192. 10.1111/ane.12192 24127647

[8] Okuyaz S Ipek R Ögenler O Yildirim DD Okuyaz Ç. Beliefs and behaviors of patients' relatives towards childhood epilepsy in Turkey. Seizure. (2022) 100:8–14. 10.1016/j.seizure.2022.05.023 35687963

[9] Singh S Mishra VN Rai A Singh R Chaurasia RN. Myths and superstition about epilepsy: a study from North India. J Neurosci Rural Pract. (2018) 9:359–62. 10.4103/jnrp.jnrp_63_18 30069092 PMC6050780

[10] Sunmonu TA Afolabi OT Komolafe MA Ogunrin AO. Patients' knowledge about their disorder: perspective of patients with epilepsy in a tertiary health facility in southwestern Nigeria. Epilepsy Behav. (2011) 20:556–60. 10.1016/j.yebeh.2010.12.030 21277835

[11] Kritz M Makinwa-Adebusoye P Du F. Women's decision-making inputs, spousal agreement and family planning in Nigeria. In: Annual Meeting of the Population Association of America. San Francisco, CA (1995).

[12] Malmusi D Vives A Benach J Borrell C. Gender inequalities in health: exploring the contribution of living conditions in the intersection of social class. Glob Health Action. (2014) 7:1–9. 10.3402/gha.v7.23189 24560257 PMC3927744

[13] Honari B Homam SM Nabipour M Mostafavian Z Farajpour A Sahbaie N. Epilepsy and quality of life in Iranian epileptic patients. J Pat Report Outcomes. (2021) 5:16. 10.1186/s41687-021-00292-3 33511464 PMC7843789

[14] Mameniškiene R Guk J JatuŽis D. Family and sexual life in people with epilepsy. Epilepsy Behav. (2017) 66:39–44. 10.1016/j.yebeh.2016.10.012 28025177

[15] Tedrus GMAS Fonseca LC Pereira RB. Marital status of patients with epilepsy: factors and quality of life. Seizure. (2015) 27:66–70. 10.1016/j.seizure.2015.02.028 25891930

[16] Chin JH. Epilepsy treatment in sub-Saharan Africa: closing the gap. Afr Health Sci. (2012) 12:186–92. 10.4314/ahs.v12i2.17 23056026 PMC3462534

[17] Mbuba CK Ngugi AK Newton CR Carter JA. The epilepsy treatment gap in developing countries: a systematic review of the magnitude, causes, and intervention strategies. Epilepsia. (2008) 49:1491–503. 10.1111/j.1528-1167.2008.01693.x 18557778 PMC3573323

[18] Ogunniyi A Osuntokun BO. Effectiveness of anticonvulsant therapy in the epilepsies in Nigerian Africans. East Afr Med J. (1991) 68:707–13. 1797533

[19] Ikeda A. Functional Mapping in Presurgical Evaluation in Epilepsy Surgery. (2010). p. 30.

